# Effectiveness of a structured circuit class therapy model in stroke rehabilitation: a protocol for a randomised controlled trial

**DOI:** 10.1186/s12883-015-0348-7

**Published:** 2015-06-10

**Authors:** Isa U. Lawal, Susan L. Hillier, Talhatu K. Hamzat, Anthea Rhoda

**Affiliations:** Department of Physiotherapy, Faculty Community and Health Sciences, University of the Western Cape, Private Bag X17, Bellville, 7535 South Africa; International Centre for Allied Health Evidence, School of Health Sciences, University of South Australia (City East), Adelaide, 5000 Australia; Department of Physiotherapy, Faculty of Clinical Sciences, College of Medicine University of Ibadan, Queen Elizabeth Road, Private Mail Bag 5017, GPO Dugbe, Ibadan, Nigeria; Department of Physiotherapy, Faculty of Allied Health Sciences, College of Health Sciences, Bayero University, Kano, Private Mail Bag 3011, Nigeria

**Keywords:** Stroke, ICF model, Circuit class therapy, Exercise intensity, Neuro-rehabilitation

## Abstract

**Background:**

Currently, the key advocacy in neuroscientific studies for stroke rehabilitation is that therapy should be directed towards task specificity performed with multiple repetitions. Circuit Class Therapy (CCT) is well suited to accomplish multiple task-specific activities. However, while repetitive task practice is achievable with circuit class therapy, in stroke survivors repetitive activities may be affected by poor neurologic inputs to motor units, resulting in decreases in discharging rates which consequently may reduce the efficiency of muscular contraction. To accomplish multiple repetitions, stroke survivors may require augmented duration of practice. To date, no study has examined the effect of augmented duration of CCT in stroke rehabilitation, and specifically what duration of CCT is more effective in influencing functional capacity among stroke survivors.

**Methods/design:**

Using a randomised controlled trial with blinded outcome assessment, this study is aimed at determining the effectiveness of structured augmented CCT in stroke rehabilitation. Sixty-eight stroke survivors (to be recruited from a tertiary health institution in Kano, Northwest, Nigeria) will be randomised into one of four groups: three intervention groups of differing CCT durations namely: 60 min, 90 min, and 120minuntes respectively, and a control group. Participants will take part in an 8-week structured intensive CCT intervention. Participants will be assessed at baseline, post-intervention, and six-month follow-up for the effectiveness of the varied durations of therapy, using standardised tools. Based on the WHO-ICF model, the outcomes are body structure/function, activity limitation, and participation restriction measures.

**Discussion:**

It is expected that the outcome of this study will clarify whether increasing CCT duration leads to better recovery of motor function in stroke survivors.

**Trial registration:**

Pan African Clinical Trial Registry (PACTR): PACTR201311000701191

## Background

Stroke is a growing global health-care crisis, with grave and disabling consequences [1]. In most countries, stroke is the second or third most common cause of death, and one of the main causes of acquired adult disability [1, 2]. Motor impairments (of upper and lower extremities) are the major recognisable impairments caused by stroke, which are associated with limitations or decline in independent mobility [3–6].

Substantial evidence suggests that task-specific training can assist functional recovery in stroke rehabilitation, with the goal of achieving true recovery of function based on motor learning principles, including purposefulness, multiple repetitions, and intensified activity [7, 8]. Circuit Class Therapy (CCT) is a form of Task Specific Training (TST) that involves the practice of structuring tasks in a circuit or series of workstations. It satisfies the three key characteristics of an effective and efficient skill training programme [9] including: (i) using different workstations that allow people to practice intensively in a meaningful and progressive way to suit their respective needs; (ii) efficient utilisation of therapists’/trainees’ time; and (iii) it encompasses group dynamics such as peer support and social support [9, 11]. Several research trials have shown that CCT is effective in improving balance, transfers, gait, gait-related activities (such as climbing stairs) and upper limb functions in stroke survivors [12, 13], especially when applied within the first six months after stroke [12, 14–16] and even later [10, 13, 17–20].

The goal of CCT in stroke rehabilitation is to institute an enduring motor learning in order to optimise motor and functional recovery necessary for the achievement of community reintegration of stroke survivors. To accomplish sustained motor learning, rehabilitation must be geared towards a relatively permanent behavioural change, which is currently believed to manifest as a result of neuroplastic change in the brain itself [21]. Compelling evidence from neuroscientific studies suggest that neuroplastic changes in the cerebral cortex and in other parts of the central nervous system (CNS) are the physiological mechanism for effective motor skill retraining following stroke [22–26]. These studies identified TST and intensity of multiple repetitions as critical nexuses to enhancing neural reorganisation and “rewiring” in the CNS. By implication the damaged brain will therefore benefit from repeated sensorimotor inputs (efferent-afferent feedback loops) in order to remodel effectively for the attainment of motor/functional recovery in stroke survivors. This signifies the need for rehabilitation professionals to focus on meaningful, repetitive, and intensive specific tasks during a rehabilitation session [27].

Stroke survivors demonstrate poor activity tolerance [28] and performance [29]. These may suggest the need for longer duration to tolerate and perform repetitive activities. The need to augment the duration of therapy in CCT for stroke survivors can be considered based on pathophysiologic and clinical domains. Pathophysiologically, the sequelae of an upper motor neuron lesion result in hemiparesis/hemiplegia, marred balance and coordination and decreased proprioceptive feedback [30]. These put together will negatively affect daily activities and exercise performance, leading to activity intolerance, increased energy cost of activity, and a decline in overall performance after stroke [27]. Cumulatively, these factors result in longer reaction time and longer time to accomplish tasks, thus suggesting the need to give adequate time for the performance of multiple repetitive tasks in stroke survivors, well beyond age and gender-matched individuals without history of stroke. Four systematic reviews and one Cochrane review have shown that augmentation of exercise therapy and/or time of exercise therapy results in significant small to moderate gains in ADL, walking ability and walking speed [31–34].

Clinically, in CCT, participants are exposed to multiple progressively structured tasks to be accomplished within a session, and considering the pathophysiological challenge of stroke survivors, they may need more time to perform multiple repetitions to enable neuroplastic changes. Rose et al. [35] have proposed that planning the contents of a session in advance with predetermined progression of tasks may allow more time for in-session practice.

In summary, pathophysiological and clinical factors may in isolation or collectively support the need to examine the effect of augmented therapy time in CCT. However, while there is a need to investigate the effect of augmenting the duration of therapy, it is equally imperative to determine how acceptable these CCT durations are among stroke survivors.

### Objective

The objective of this study is to investigate the relative effectiveness of augmented durations of CCT on the functional capacity of stroke survivors. Additionally, the study will investigate the effectiveness of augmented duration of CCT on upper and lower extremity functions (with respect to body structure/function, activity limitation and participation restriction) post-stroke and the acceptability of the various CCT durations among stroke survivors. For these to be achieved outcomes of interest will be assessed based on the World Health Organisation International Classification of Function, Disability and Health model (WHO-ICF) [36].

## Methods/Design

### Study design/setting

This study is a randomised controlled trial of the effectiveness of augmented durations of a structured CCT model in the rehabilitation of stroke survivors. All participants will be recruited from Aminu Kano Teaching Hospital (AKTH) in Kano State, Northwest of Nigeria. The hospital (AKTH) is a tertiary health institution, situated in Kano, the most populous state in Nigeria, with over 9 million inhabitants [37]. It is a 500-bed-capacity hospital that receives patients from within Kano and the neighbouring states of Jigawa, Katsina, Kaduna, Bauchi and Zamfara states. The patronage list comprises primarily the indigenous Hausa Fulani tribe, although Nigerians of other tribes such as the Ibo and Yoruba ethnic groups also constitute a sizeable number of the clientele.

### Participants

Participants will include all stroke survivors in AKTH referred for physiotherapy by consulting physicians. However, only participants who meet the inclusion criteria will be considered for randomisation into the study groups (involving intervention and control groups). Participants will be considered eligible if: stroke is ascertained to be due to cerebrovascular accident; leading to unilateral motor deficits [14]; they provide a written informed consent; they are adult stroke survivors of ≥18 years of age; onset of stroke is ≥30 days; they possess sufficient cognition to participate (having a score of ≥24 points on mini-mental state examination); they are willing to participate in an 8-week intensive CCT programme; have the ability to walk for 10 m unsupported (walking aid is allowed); and have a minimum active wrist extension (2/5 on manual muscle testing). Participants will be considered ineligible if they present with precluding medical comorbidity to exercise, and history of any major surgical procedure significant enough to interfere with performance (general or orthopaedic) in an exercise therapy intervention.

Stroke survivors who meet the eligibility criteria will be randomised into one of four arms of the study, including three intervention categories (60 min CCT, 90 min CCT and 120 min CCT) and one control (60 min standard physiotherapy).

### Sample size and power calculation

Using power calculations to detect a between-group difference of 42.5 m (0.43 effect size) for a 4-group repeated measure MANOVA in walking distance with 90 % power at α = 0.05, a total sample size of 56 was generated, using an estimated standard deviation (SD) for the calculation as adopted from a meta-analysis [9]. The 42.3 m was proposed as the minimum clinically important difference in walking distance, based on previous studies of implicit measurement error following repeated measurement of speed. The generated total sample size of 56, by implication, will give 14 participants as samples for each group. To incorporate drop-out to follow-up, we hope to recruit a total of 68 participants (17 participants per group). The power calculation was conducted using G*Power version 3.0.10.

### Randomisation and blinding

**Fig. 1:**
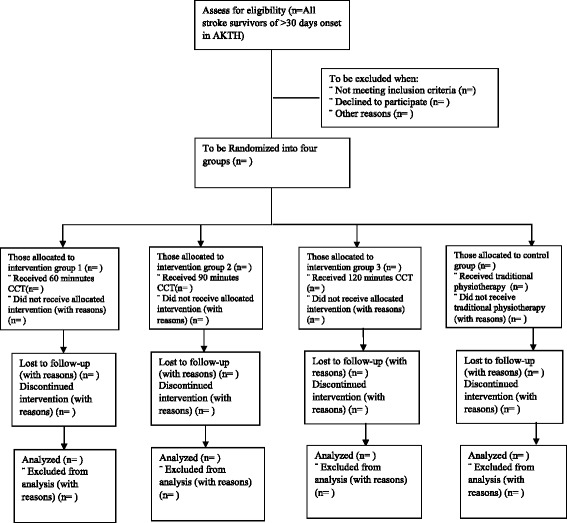
Outline of study flow diagram

Randomisation will be conducted using a computer-generated random allocation sequence schedule held by a third party, who will randomly allocate recruited participants into the study group (Fig. 1. outline of the study flow diagram based on CONSORT [38]). To eliminate bias, the assessment of outcome will be performed by (experienced/trained) blinded assessors, who will be blinded to the nature/type of intervention as well as the intervention groups of the participants. Participants will also be instructed not to disclose their individual intervention groups to the assessors.

### Procedures

This study has been approved by both the Senate Research Grants and Study Leave Committee University of the Western Cape (South Africa) (ethics number 13/9/33) and the Human Research Ethics Committee of Aminu Kano Teaching Hospital (Nigeria) (NHREC/21/082008/AKTH/EC/1232).

Assessment of participants will be conducted at three stages (baseline, post-intervention, and at 6-months follow-up). To ensure a comprehensive assessment, we chose a battery of measures covering the WHO-ICF model [36]. We selected certain tools to cover the three key domains proposed by the ICF: body structures and function, activity and participation. At baseline, participants will be assessed for socio-demographic characteristics which will include personal demographic information and stroke-specific information. The personal demographic information will include age, sex, height, weight, marital status (pre- and post-stroke), educational qualification, employment (pre- and post-stroke), and tribe. The stroke-specific information will include time since stroke, type of stroke, hemispheric side of lesion, and use of cane. This will be followed by ICF-based assessments, all at baseline. The outcome measures to be employed for these assessments and their function/application are presented in Table 1.

**Table 1:** Study assessment tools

Scales	Function/application
Body structure and function assessment	
i. Modified Tardieu Scale (MTS)	
	The MTS measures spasticity [39]. Descriptively, the MTS has two measurements, the quality of muscle reaction (ordinal scale) and the angle of reaction or angle of catch (ratio). The quality of muscle is scored from 0–5; 0 implies no resistance to Passive Range of Movement (PROM) and 5 indicating joint immobile. On the other hand Angle of catch can be understood via two factors of PROM, the speed of movement and joint angle. The reporting of MTS summarily, involve the quality of muscle and angle of reaction components making it to fit into the body structure and function of the ICF absolutely. It has excellent test retest reliability (ICC = 0.86) in stroke patient [40], with good convergent validity for both elbow and ankle joints (*r* = 0.86 and *r* = 0.62 respectively) [39].
ii. Medical Research Council Manual Muscle Testing (MMT)	
	MMT will be used to assess muscle strength for upper and lower extremities, attention will be paid to specific joints of both extremities. For the upper extremity attention will be focused on shoulder, elbow and wrist joints and for the lower extremity joints hip, knee and ankle will be measured. MMT is the best known and most frequently employed muscle strength grading system for manual muscle testing (MMT) [41]. It has a score range of 0–5, with 0 being the minimum and 5/5 the maximum. An excellent test-retest reliability for both right and left hip joints (ICC = 0.98 and ICC = 0.97 respectively) with osteoarthritis [42]. Its convergent validity ranges between adequate to excellent in different body parts [43].
Activity assessment	
i. Modified Rankin Scale (MRS)	
	The MRS is a hierarchical scale of 0–6 points that indicate “global disability”. It is the most prevalent functional outcome measure for stroke research. Lower scores on the scale suggest more independence and higher scores signify increased dependency. Its test-retest reliability ranged between adequate to excellent (Kappa = 0.67-0.96) [44], with an excellent convergent validity [45].
ii. Modified Barthel Index (MBI)	
	The MBI assesses ten functional tasks of daily living (activities of daily living – ADL). It scores the individual based on independence in each task. Scores range from 0 and 100, with a higher score indicating greater independence. The inter-rater reliability is sufficient at the item level (kappa 0.50–0.78) and good for the overall inter-rater agreement (intraclass correlation coefficient [ICC] 0.77) [46, 47].
iii. Six-minute Walk Test (6MWT)	
	6MWT is a clinically useful measure of walking ability post stroke, which incorporates the important requirements of ambulation, such as walking speed, dynamic balance, and submaximal endurance. It is performed at the individually determined fastest speed possible during walking, making it ideal for stroke survivors [48]. It measures an individual’s ability to walk for a maximum distance (meters) within 6 min. This test exhibits excellent test-retest reliability (ICC = 0.973; 95 % CI = 0.925 to 0.988), a minimal detectable change of 54.1 m, and an acceptable concurrent validity (*r* = 0.52 to 0.89) [48].
iv. 10 Meter Walk Test (10MWT)	
	Participants’ gait speed will be measured using 10MWT [48], which will be calculated by the time required to cover a distance of 10 m. Participants will be asked to walk at their maximal speed using their regular foot wear and walking aids (for those who use aids). The test will be performed on a 14 m walkway, to avoid the effects of acceleration and deceleration, therefore the individual may accelerate 2 m before entering the 10 m distance and 2 m to decelerate afterward, this will ensure a steady velocity within the 10 m mark. 10MWT shows a high intra-observer reliability (ICC = 0.95) and validity (*r* = 0.79) in stroke survivors [49].
v. Action Research Arm Test (ARAT)	
	The ARAT is a criterion-rated assessment of upper extremity activity limitations [50]. The ARAT includes 19 items divided into four subscales: grasp, grip, pinch, and gross movement. The items within each subtest are ranked based on a four-point ordinal scale ranging from zero to three, where three symbolises normal performance on each item. The items are ordered in a hierarchy, allowing skipping some items if the person is unable to do an earlier item normally. A score of 57 indicates normal performance. The test has a good test-retest reliability for both chronic and acute stroke, ICC = 0.963 [51], internal consistency α = 0.985 [50] and construct validity in relation to the arm section of Fugl Meyer, ICC = 0.925 [51].
vi. Motor Activity Log (MAL)	
	The motor activity log (MAL) is a rating scale that evaluates how the affected hand is used to perform 30 daily activities (e.g., feeding, turning a door handle). For each activity, the patient rates how much the affected hand is used (amount of use, AOU) and how well the activity is performed (quality of movement, QOM). Ratings are usually on a scale of 0 to 5, with higher scores representing better functions. Scores on each scale are calculated as the mean of the scored items attempted with the affected arm. Its internal consistency is good, α > 0.81, with acceptable test retest reliability *r* > 0.91 and stability ratio >3 for the QOM and AOU, though not found to be reliable [52].
Participation assessment	
Stroke specific Quality of Life Questionnaire (SS-QOL)	
	SS-QOL is selected to assess community participation. The SS-QOL is a self-report questionnaire consisting of 49 items cutting across 12 domains of mobility, energy, upper extremity (UE) function, work/productivity, mood, self-care, social roles, family roles, vision, language, thinking, and personality specific for stroke survivors. The domains are graded individually, and a total grade is also rendered [53]. SS-QOL has a good content validity, kappa coefficient ranged from 0.75-1.00, it demonstrated multiple representations of the ICF categories and covered a broad range of the ICF components that were meaningful for the stroke subjects [54].
Acceptability	
	To assess acceptability participants will complete a purpose-designed questionnaire [55]. The tool is a six-item scale adapted from the original treatment acceptability questionnaire, it is a seven point scale, with lower score indicating lower acceptability. Possible score on the scale ranged from 6–42. Participants in all the intervention groups and the control will be asked to provide information specific to their treatment. The test has not been tested for reliability and validity.

All baseline measures will be repeated immediately post-intervention and 6 months follow-up, excluding descriptive personal and stroke data. Also at this time, the intervention acceptability measure will also be applied.

Participant adherence will be duly monitored and recorded for each session in terms of attendance (number of sessions) and amount of practice (within sessions – time spent and repetitions where relevant). Fidelity of the intervention will be monitored by the primary investigator performing video recordings of randomly chosen sessions in each arm. Co-investigators will review these videos for compliance with the established practice protocols. Safety issues and adverse events will be recorded by treating staff in each group and monitored by the co-investigators. Previous trials using CCT have found no increase in adverse events as compared to usual care [32].

### Intervention groups

The intervention groups are the three intensities (durations) of CCT namely 60 min, 90 min, and 120 min, tagged groups A, B and C respectively. All participants will be assessed at baseline (prior to the intervention), post-intervention and at six-month follow-up. A total of 10 workstations will be made available in the circuit, arranged to progress in complexity. These stations will be made up of task-specific activities for the upper and lower extremity, structured alternately across the circuit (i.e., after every lower extremity workstation an upper extremity workstation follows), ensuring a 1:1 ratio of upper to lower extremity activities. This is to allow for adequate concentration, specificity of activity choice, and distribution of equal activity duration for both upper and lower extremities. A minute change period (not within specified duration of intervention) will be allowed for crossing from one workstation to the next.

The intervention is an 8-week, 3-times weekly training programme, giving a total of 24 sessions. Activities will be individualised allowing each participant to perform at a level based on his/her ability, and progress steadily within the allotted time for each group.

The upper extremity task-orientated CCT activities will include activities to improve fine motor skills, grasp and reach, sensory function, and proximal control. Similarly, tasks for the lower extremity will be targeted to balance, strength, cardiovascular endurance, and retraining of gait mobility. All CCT sessions will be conducted by three trained physiotherapists, with each treatment session structured at a 3:1 ratio of patients to therapists. Table 2 (below) presents the CCT task-specific activities to be implemented in this study.

**Table 2:** Circuit class therapy task specific activities for the intervention

Stations/description	Prescribed tasks
Workstation 1	
Tasks for warm-up specific for upper extremity	
	Active flexion- extension of shoulder, elbow and wrist joints
	Abduction-adduction of shoulder joint
	Upper extremity weight bearing on physiotherapy ball
	Push-ups on physiotherapy ball or using chair arm rest
Workstation 2	
Tasks for warm-up specific for lower extremity	
	Stretching the lower extremity (flexion/extension of the limb in supine or sitting position)
	Marching on spot
	Shuttle walking
	Jogging on spot
Workstation 3	
Tasks to achieve reaching, gripping and transferring light objects	
	Sitting with arm supported on high plinth at 90° shoulder flexion
	Active protraction to push small objects (light ball) off edge of plinth to target the wall
	Sitting same way to push weighted object (a heavier ball)
	Active horizontal abduction and adduction to reach object (cup) on the wall
	Use of protracted shoulder to open a door with patient standing three feet away from the door
	Wrist flexion/extension in gravity counter balance (provide a target to aim for)
	Radial and ulnar deviation (in gravity counter balance) with a target to push (cup)
	Picking light objects from table to the wall and back
Workstation 4	
Tasks to achieve lower extremity flexibility and function	
	Timed shuttle walk/Initiation of minimal Shuttle jogging (50 % of time allotted for this station)
	Sit to stand from high chair with arm rest (placing affected leg behind the intact)
	Stationary bike riding
	Squatting activity using the wall bars
Workstation 5	
Task to achieve upper extremity strength/control	
	Active shoulder flexion, extension and abduction with weight of varying sizes (dumbbells)
	Active shoulder flexion, extension and abduction with resistant band to reach for a target on the wall (cup)
	Active shoulder abduction with weights of varying sizes (dumbbells), to reach for a target on the wall
	Active elbow flexion/extension with resistance band. Also substitute with varying weights
	Active wrist flexion/extension, ulna/radial deviations with resistance band. Can be substituted later with weights of varying sizes
	Finger to nose movement
	Rapid hand alternating movements
Workstation 5	
Task to achieve upper extremity strength/control	
	Active shoulder flexion, extension and abduction with weight of varying sizes (dumbbells)
	Active shoulder flexion, extension and abduction with resistant band to reach for a target on the wall (cup)
	Active shoulder abduction with weights of varying sizes (dumbbells), to reach for a target on the wall
	Active elbow flexion/extension with resistance band. Also substitute with varying weights
	Active wrist flexion/extension, ulna/radial deviations with resistance band. Can be substituted later with weights of varying sizes
	Finger to nose movement
	Rapid hand alternating movements
Workstation 6	
Task to achieve balance/coordination while walking	
	Sit to stand from lower chair without arm rest (affected leg behind a distant placed intact leg)
	Standing on foam eye closed (safety is key in this activity)
	Carrying object while on shuttle walking (a tray with cup of water)
	Walk up and down stairs (patient walk backward while coming down stairs)
	Sudden stops and turns while walking
	Obstacle crossing while walking
	Figure 8 walking
Workstation seven	
Task to achieve improved grip, precision and dexterity with upper extremity	
	Draw a line on the white board
	Rolling a dumbbell forwards and backwards on a flat surface (table)
	Open and close a window
	Take lids off bottles
	Bring object from table to mouth, vary size and weight of objects
	Pour water from jug to cup
	Mix water with spoon of various sizes
	Take money in and out of a purse
	Fold paper and place in an envelop
	Trace pattern of different figures on white board
Workstation 8	
Task to achieve lower extremity strength/control of gait	
	Walking different step length of parallel line
	Obstacle crossing while walking with a tray of cups filled with water
	Walking backward and side ways
	Heel lift in standing without and with carrying an object
	Walk on toes short distance forward and backward
Workstation 9	
Task to achieve advanced motor task with upper extremity	
	Rolling pin pushing forward and backward
	Reach, grasp and move objects to and from different heights
	Wipe over windows
	Wash, wring and peg clothing on lining rope
	Paint sketched objects on cardboard paper
	Use key boards to type
	Cut customised foams using knifes of varying sizes
	Put-on common clothing and foot wears
Workstation 10	
Tasks to achieve improved outdoor activities with lower extremity	
	Walking while picking objects from the floor
	Walking through closely packed obstacles
	Walking through tight space
	Jumping on foam eyes closed
	Walking on joined foams
	Reverse walking on straight line
	Treadmill walking/jogging
	Speed stair climbing
	Outdoor walking
NOTE:	
1. Tasks within stations are not necessarily convenient and possible for all participants, choice is therefore individualized	
2. Tasks in stations vary because it is not necessary for participants to undergo all activities and also to allow room for wide range of opportunities and choices	
3. Progression might be based on activities not initially possible within stations or based on modifications considered necessary by physiotherapist	
4. Tasks are performed as structured in the model based on durations allotted for each group	

Adapted from circuit class therapy intervention manual version 1.0 [56]

### Control group (standard physiotherapy)

The standard physiotherapy group, like the intervention groups, will involve the same number of sessions (24), duration (60 min) and frequency per week (3) of therapy. Standard physiotherapy will comprise one-to-one therapist/patient sessions engaging in impairment-centred mobilisation techniques, standing balance (using varying methods) and functional activities for both upper and lower extremities. All the activities for the control group will be implemented by regular therapists (who are similar in qualification/experience to therapist implementing the CCT programme) in the Physiotherapy Department of AKTH.

### Data analysis

Data will be recorded in Microsoft Excel before being exported to Statistical Package for Social Science (SPSS). Both descriptive and inferential statistics will be used to examine the outcomes of the study.

Descriptive statistics of frequencies, percentages, mean, and standard deviation will all be used to describe baseline assessments and demographic characteristics of participants. Where appropriate, they will also be used at post-intervention and follow-up to describe relevant findings.

Between-groups relative mean differences of the dependent variables, (spasticity, muscle strength, functional independence, ADL, functional capacity, gait speed, upper extremity function and impairment) will be determined using the general linear model repeated measures, MANCOVA models (for each study domain, adjusted central on baseline as covariates). If the MANCOVA is found to be significant (Roy’s largest root), univariate between-groups results will be reported and pair-wise post hoc analysis will be performed using least significant difference.

A multiple regression analysis will be performed to investigate the relation between improvement at different time periods of CCT and performance in each of the specific dependent variables, to detect which of the durations most effectively predicts improvement in such a variable.

## Discussion

Augmenting exercise therapy time to improve recovery outcomes has been supported by some research findings [31–34]. However, the amount of augmented time is not known, using CCT as a delivery model. In this era of evidence-based practice, there is an urgent need to support all facets of implementing CCT with cogent evidence prior to adoption. The *need* to augment the duration of therapy in stroke survivors might not be challenged, but exactly *what* duration (intensity) is suitable, in terms of acceptability and therapeutic benefit to the stroke survivor, needs to be supported empirically.

Reporting the outcome of this study using the ICF model will provide an opportunity to identify which of the domains – body structure/function, activity or participation – will be more responsive to change following intervention, and to which specific duration of intervention.

It is envisaged that engaging in task-specific upper limb practice and task-specific practice involving the lower limb will add credence to this study in explaining which section of these body parts will be functionally better responsive to a particular augmented duration of CCT.

The fact that this study is taking place in a minimally resourced country, will further support the cost benefit of CCT in any setting, and will globalise the empirical findings for CCT.

Finally, if the outcomes of this study support a specific duration of CCT, it will serve as a guide for clinical recommendations in CCT implementation.

## Abbreviations

CCT: Circuit class therapy
TST: Task specific training
CNS: Central nervous system
PACTR: Pan African clinical trial registry
WHO: World Health Organization
ICF: International classification of function
AKTH: Aminu Kano teaching hospital
ADL: Activities of daily living
SPSS: Statistical package for social sciences
MTS: Modified Tardieu Scale
MMT: Manual muscle testing
MRS: Modified rankin scale
MBI: Modified Barthel Index
6MWT: Six-minute walk test
10MWT: 10 Meter walk test
ARAT: Action research arm test
MAL: Motor activity log
SS-QOL: Stroke specific quality of life questionnaire
